# Occupational Health in India

**DOI:** 10.29024/aogh.2302

**Published:** 2018-08-31

**Authors:** Rajat Kumar Saha

**Affiliations:** 1 Hero MotoCorp Ltd, Dharuhera, Haryana, IN

## Introduction

Occupational health is defined as the highest degree of physical, mental and social well-being of workers in all occupations. It is the branch of healthcare which deals with all aspects of health and safety at the workplace. It lays strong emphasis on the prevention of hazards at a primary level. Occupational health is essentially preventive medicine [1]. Consider the following facts from India: total population is 1.324 billion (2016); gross national per capita income (PPP) is 6490$; life expectancy at birth (Male/Female) is 67.3/69.8 years; probability of dying under the age of five (per 1000 live births) is 48; total expenditure on health per capita is 75$; and total expenditure on health as a percentage of GDP is 4.7 [2].

## Occupational Health Legislation in India

There are presently 16 laws related to working hours, conditions at work and employment. There are two acts containing the main provisions for legal measures for the protection of health and safety of workers; they are the Factories Act (1948) and the Mines Act (1952). The Factories Act was amended in 1987 and stipulates pre-employment examination as a pre-placement procedure, statutory periodic medical examination for job in hazardous areas. In India, occupational health is under two ministries: 1) Labour and 2) Health and Family Welfare. The Ministry of Labour and the labour departments of the states and union territories are mainly responsible for health and safety of workers. The Ministry of Health and Family Welfare is responsible for providing health and medical care to workers through its facilities. The DGMS (Directorate General of Mines Safety) and the DGFASLI (Directorate General – Factory Advisory Services and Labour Institutes) assist the Ministry in technical aspects of occupational health and safety in mines, ports and factories respectively.

### Constitutional Provisions

There are three articles for ensuring workers’ safety and health. Article 24 prohibits employment of children under the age of 14 years. Article 39 (e and f) states that the health of men, women and children should be protected, and children should be given opportunity and facility for healthy development and should be protected against exploitation. Article 42 states that humane conditions at work and maternity relief should be provided.

## Occupational Health Institutions

The National Institute of Occupational Health (NIOH) was established in 1970 at Ahmedabad, Gujarat, as a WHO collaborative and reference centre for occupational health, and it works closely with the Ministries of Labour, Health and Family Welfare, Environment and Forests, Agriculture etc. [3]. Some thrust areas of the Institute are: occupational and environmental epidemiology, toxicology, environmental pollution, women’s health, agricultural health and human resource development. The objectives of NIOH are: to promote intensive research to evaluate environmental stresses/factors at the workplace, to promote the highest quality of occupational health through fundamental and applied research, to develop control technologies and health programmes through basic and fundamental research and to generate human resources in the field. Two Regional Occupational Health Centres (ROHCs) have been set up in Bangalore and Calcutta. The National Safety Council of India (NSCI) was established to promote safety consciousness among workers to prevent accidents, minimise dangers and risks and arrange related education and awareness programmes. The three main activities of the NSCI are: road transportation safety; safety of health in the construction sector; safety, health and environment in small – to medium-scale enterprises (SMEs). Other public institutes include the Central Labour Institute (and its associated institutes) and the All India Institute of Hygiene and Public Health. The Indian Association of Occupational Health (IAOH) is an association of over 3000 members comprising health professionals, industrial hygienists, safety professionals, social workers and others. It aims to promote occupational health by various measures including conducting training courses, workshops and conferences, producing a journal with scientific articles, conducting research activities, collaborating with international agencies in the field and preparing a national registry of occupational health.

## Occupational Health Statistics (India)

National Institute of Miners’ Health (NIMH), an autonomous Institute under the Ministry of Mines, Government of India, conducts applied research in occupational health and hygiene and specializes in providing technical support services to mining and mineral-based Industry with special reference to the metalliferous sector and endeavours for safe mines and healthy miners through research and development. As per NIMH, the prevalence of pneumoconiotic opacities in chest radiographs in open cast mine workers in 2005 and 2011 were 5.7% to 12% and 5.3% to 13%, respectively [4]. In 2011, out of 101 workers in a stone mining area suffering from respiratory diseases, 73 suffered from silicosis, of whom 16 had silicosis with progressive massive fibrosis (PMF). A survey conducted in an underground metal mine has shown that almost 75% of mine workers had evidence of noise-induced hearing loss. In a recent survey conducted by NIMH in various mines, out of 117 HEMM (Heavy earth moving machinery), 100% dozers, 95% loaders, 90% dumpers and tippers, 15% excavators and 8% shovellers showed moderate to high health risks to operators due to whole-body vibrations. Of 48 HEMM operators, 85% complained of various musculoskeletal disorders related to back, shoulder, neck and knees. In India, major occupational diseases are pneumoconiosis (including silicosis, bagassosis, anthracosis and byssinosis), asbestosis, other chronic lung diseases, musculoskeletal injuries, noise-induced hearing loss, pesticide poisoning and accidents. Occupations related to construction, mining and agriculture have high levels of related diseases. Occupational health nurses are the largest single group of health professionals involved in delivery of health services at the workplace. They are at the front line in helping to protect and promote the health of working population [5]. The concept of occupational health nursing is new to India. It is non-existent in unorganised sectors. Even the public sector and private employers have not yet realized its importance. There is a need to create awareness about this issue amongst all stakeholders.

## National Policy on Occupational Health

The Ministry of Labour and Employment, Government of India, approved the national policy on safety, health and environment at workplaces in February 2009 [6]. It provides guidelines for developing and maintaining safety culture and environment at workplaces for all stakeholders. It also deals with provision of a statutory framework, administrative and technical support services, providing incentives (both financial and non-financial) to employers and employees, developing research and development capabilities, prevention strategies and their monitoring and providing required technical manpower and inclusion of safety, health and environment improvement in other national policies.

An action programme for policy implementation is part of the documented policy. It includes eight specific working areas for action – enforcement, development of national standards, ensuring compliance, increasing awareness, promoting research and development, occupational safety, health skills development and data collection. The government of India is committed to implementing the national policy on safety, health and environment at workplaces through tripartite consultations and mobilisation of resources and expertise of all concerned stakeholders. The guidelines of the policy are helpful since it envisages total commitment and demonstration by all concerned stakeholders such as governments and social partners through well-set goals and objectives. Through dedicated and concerted efforts, India will steadily march towards economic prosperity consistent with the requirements of OSHE, thereby improving the people’s standards of living.

A policy review was done initially to determine the status of health, safety and environment at the workplace. Subsequent reviews of the policy and action programme is planned for once in five years. Assistance was taken from the report of the working group on occupational safety and health for the 11th and 12th five-year plans under the Ministry of Labour and Environment, Government of India. As per the data available, the number of working factories for the years 2003 to 2007 has increased by about 46% with the increase in average daily employment from 4.92 million to 8.02 million. The number of injuries also decreased by about 7% i.e. from 16,432 to 15,290; however, the fatalities during the period increased from 525 to 821. It may be noted that the frequency rate of injuries significantly reduced by about 30% during the above period. The number of reportable accidents in major ports from 2003–2007 decreased from 191 to 158 thus registering a decrease of about 17%. The number of fatal accidents also decreased from 29 to 23, thus registering a decrease of about 20% during the same period. Significant data on five yearly reviews of policy could not be gathered.

### National Programme for Control and Treatment of Occupational Diseases

Occupational health was one of the components of the National Health Policy in 1983 and 2002. The Ministry of Health and Family Welfare, Government of India, launched a programme entitled “National Programme for Control and Treatment of Occupational Diseases” in 1998–99. The National Institute of Occupational Health, Ahmedabad, is the nodal agency for the same. The categories of major occupational diseases in India are: [7] occupational injuries, occupational lung diseases, occupational cancers, occupational dermatoses, occupational Infections, occupational toxicology and occupational mental disorders.

A grouping of major occupational disorders in India according to the etiological factors includes – occupational injuries: ergonomics related; chemical occupational factors: dust, gases, acid, alkali, metals etc.; physical occupational factors: noise, heat, radiation etc.; biological occupational factors; behavioural occupational factors; and social occupational factors.

In India in 1998–99, the prevalence of silicosis was 6.2–34% in mica miners, 4.1% in manganese miners, 30.4% in lead and zinc miners, 9.3% in deep and surface coal miners, 27.2% in iron foundry workers, and 54.6% in slate-pencil workers. Prevalence of asbestosis was extended from 3% in asbestos miners to 21% in mill workers. In textile workers, byssinosis was as common as 28–47%. Nutritional status in terms of body mass indices (BMI) of the workers was also significantly low.

### National List of Occupational Diseases in India

As per The Indian Factories Act 1948 3rd Schedule, [8] Sections 89 and 90 – list of notifiable diseases, there are 29 enlisted diseases. They include poisoning by metals and compounds such as lead, tetra-ethyl lead, phosphorous, mercury, manganese, arsenic, nitrous fumes, carbon bisulphide, benzene, their nitro or amido derivatives or its sequelae, chrome ulceration, anthracosis, silicosis, radium or other radioactive substances, halogens or halogen derivatives, cancer of the skin, toxic anaemia, jaundice, oil acne or dermatitis due to mineral oils, byssionosis, asbestosis, contact dermatitis, noise-induced hearing loss, beryllium, carbon monoxide, coal miners’ pnoumoconiosis, phosgene, isocyanates, occupational cancer and toxic nephritis.

## Compensation

The Workmen’s Compensation Act, 1923, has four chapters and the following schedules: Schedule I, Parts 1 (list of injuries deemed to result in permanent total disablement) and 2 (list of injuries deemed to result in permanent partial disablement), Schedule II (list of persons who are included in the definition of workmen) and Schedule III (list of occupational diseases) [9]. As per Chapter II, Workmen’s Compensation, clause 3, Employer’s liability for compensation, (1) If personal injury is caused to a workman by accident arising out of and in the course of his employment, his employer shall be liable to pay compensation in accordance with the provisions of this Chapter.

If a workman employed in any employment specified in Part A of Schedule III contracts any disease specified as an occupational disease related to that employment or if a workman has been employed for a period of not less than six months in any employment specified in Part B of Schedule III contracts any disease specified in Part C of Schedule III for such period as the Central Government may specify, the disease shall be recognised as injury by accident and the accident shall be deemed to have arisen in the course of the employment.

Amount of compensation (1) Subject to the provisions of this Act the amount of compensation shall be as follows namely: where death results from the injury an amount equal to fifty per cent of the monthly wages of the deceased workman multiplied by the relevant factor; or an amount of fifty thousand rupees whichever is more; where permanent total disablement results from the injury an amount equal to sixty per cent of 5 monthly wages of the injured workman multiplied by the relevant factor; or an amount of sixty thousand rupees whichever is more.

## Conclusion

Though legislation is in place for ensuring adequate delivery of occupational health and safety services, with supporting national programmes, policies and institutions, still there are many lacunae which impose challenges for attaining the aims and objectives.

## Challenges

The lacunae in Occupational Health system in India can be highlighted as follows:

1. A very large proportion of the workforce is in the unorganised sector (more than 90% vs. less than 10% in the organised sector). The occupational health management system, implementation and beneficiaries are limited largely to the organised sector, even today, after years of advancements in every field.
2. Though legislation exists to protect workers, ineffective and incomplete implementation of this legislation is a major constraint.
3. Lack of trained occupational health manpower with deficient institutions, qualification courses, training modules, infrastructure, facilities and budgetary provisions make the implementation of legislation a challenge. There is low priority and spending on public health, which is reflected in the field of occupational health as well.
4. India is a densely populated nation with a high unemployment level; as such, there is ready availability of labour at lower wages. In such situations, health and safety at the workplace is often compromised.
5. A huge extent of undiagnosed and unreported occupational illnesses lead to a lack of accurate information and data on the scope and extent of occupational diseases.
6. There is indifference and apathy of employers, employees, the general public and other stakeholders to occupational health issues.
7. There is a lack of awareness about occupational health issues among all stakeholders.
8. Segregation and alienation of the occupational health discipline from primary health care and general health services is itself a challenge to reach out to the unorganised sector.
9. The concept of occupational health nursing is new to India. It is non-existent in the unorganised sector. Even the public sector and private employers have not yet realised its importance.
10. Poverty is an additional risk factor with low-income youths more likely to work in high-risk occupations such as agriculture, mining and construction.
11. Child labour, though legally committed, leads to poverty-related health problems.
12. The national policy on safety, health and environment at workplaces, which was launched in 2009, is yet to be fully implemented.

## Specific recommendations

The following are the main present and future needs in occupational health in India:

1. Existing occupational health related legislation and facilities need to be expanded and extended to workers in the unorganised sector with immediate implementation and periodic review for improvement.
2. Further development of institutions and infrastructure of occupational health, with simultaneous training of professionals in the field.
3. Spreading awareness of occupational health related issues among all stakeholders such as employers, employees, lawmakers, workers’ organisations (e.g. trade unions), non-governmental organisations (NGOs) and the general public.
4. Integration of occupational health into primary health care and general health services through the concept of BOHS (basic occupational health services).
5. There is an urgent requirement of modern occupational health and safety legislation, adequate enforcement machinery and establishment of centres of excellence in occupational medicine in all states of the country controlled by a central institute, to catch up with the global pace.
6. There is a need to increase awareness about the concept of occupational health nursing among all stakeholders along with recruitment of adequately trained occupational health nurses for implementing basic occupational health services.
7. Basic issues which are barriers to economic development as well as implementation of occupational health policy like dense population, unemployment, poverty, illiteracy, ignorance and unskilled manpower need to be addressed urgently.
8. The national policy on safety, health and environment at workplaces (2009) needs to be implemented urgently in full swing with a detailed five-year review.
